# B-type natriuretic peptide and mortality in extremely low birth weight infants with pulmonary hypertension: a retrospective cohort analysis

**DOI:** 10.1186/1471-2431-14-68

**Published:** 2014-03-11

**Authors:** Alain Cuna, Jegen Kandasamy, Brian Sims

**Affiliations:** 1 Department of Pediatrics, Division of Neonatology, University of Alabama at Birmingham, 619 S 19th St, Birmingham AL, 35249, USA

**Keywords:** Prematurity, Bronchopulmonary dysplasia, Prognostic factors, Outcome

## Abstract

**Background:**

B-type natriuretic peptide (BNP) is a strong predictor of mortality in adult patients with various forms of pulmonary hypertension (PH) and may be a strong prognostic marker in extremely low birth weight (ELBW) infants with bronchopulmonary dysplasia (BPD) associated PH as well. We sought to assess the relationship between BNP levels and all-cause mortality in a cohort of ELBW infants with BPD and PH.

**Methods:**

We retrospectively identified ELBW infants with BPD and PH who had serum BNP levels measured as part of routine clinical care in the neonatal intensive care unit. Peak serum BNP levels were correlated with survival to discharge or death.

**Results:**

Thirty-six ELBW infants (mean gestational age 26.0 ± 1.9 weeks and mean birth weight 740 ± 290 grams) with BPD and PH had available survival data and had serum BNP levels measured. Peak BNP level was significantly lower among infants who survived than among those who died (128 pg/ml, [IQR 23 to 463] vs. 997 pg/ml, [IQR 278 to 1770], P < 0.004). On multivariate Cox proportional hazard analysis, BNP predicted survival independent of age, gender, and BPD severity. Area under receiver operator characteristic analysis identified a BNP value of 220 pg/ml to have 90% sensitivity and 65% specificity in predicting mortality.

**Conclusion:**

BNP estimation may be useful as a prognostic marker of all-cause mortality in ELBW infants with BPD associated PH.

## Background

Pulmonary hypertension (PH) is increasingly recognized as an important complication of prematurity and bronchopulmonary dysplasia (BPD) [1,2]. Retrospective studies have estimated that 25 to 37% of infants with BPD develop PH [3,4], and a recent prospective study showed that 1 out of 6 extremely low birth weight (ELBW) infants develop PH [5]. This is concerning as PH in the BPD population is associated with worse outcomes, with mortality rates ranging between 14% and 38% in retrospective studies [3,4,6-8] and 12% in one prospective study [5]. Currently there are no clear guidelines for assessment and monitoring of ELBW infants with PH [9]. Identification of a widely available biomarker with strong prognostic information is highly desirable for risk stratification and management.

B-type natriuretic peptide (BNP), a cardiac biomarker released by myocytes in response to ventricular stretch [10], is an established marker of ventricular dysfunction [11]. In adult patients with PH, levels of BNP correlate with hemodynamic parameters of disease severity and has been shown to be predictive of survival [12-16]. It is possible that BNP could also prove useful in assessing severity and prognosis of ELBW infants with BPD and PH. The purpose of this present study is to evaluate the utility of BNP as a potential marker for predicting mortality among ELBW infants with BPD and PH.

## Methods

This retrospective cohort study was conducted in the Neonatal Intensive Care Units at the University of Alabama at Birmingham Hospital and Children’s of Alabama Hospital between August 2010 and December 2012. This study was approved by the University of Alabama at Birmingham Institutional Review Board, with waiver of informed consent.

### Study population

We identified from the neonatology database all ELBW infants with BPD in whom serum BNP concentrations were measured as part of routine clinical assessment for PH. Diagnosis of BPD was based on the National Institute of Health consensus definition [17]. Diagnosis of PH was based on the presence of at least 1 of the following echocardiographic findings: (1) presence of elevated tricuspid regurgitation jet, (2) flattening of intraventricular septum, (3) right ventricular hypertrophy, or (4) right to left shunting. Echocardiogram studies were performed by certified technicians using Sonos 5500 ultrasound machine (Philips Healthcare) and were independently interpreted and reported by pediatric cardiologists. Infants with structural heart disease other than a patent ductus arteriosus (PDA) or patent foramen ovale and those with multiple congenital anomalies were excluded.

### Data collection

Data were collected from the infants’ medical records. Data extracted included baseline demographic information, respiratory support at 36 weeks postmenstrual age, diagnosis and severity of BPD, other co-morbidities of prematurity including PDA, severe intraventricular hemorrhage, proven necrotizing enterocolitis, medications used for treatment of PH, and serum BNP levels. Peak BNP level was defined as the highest BNP concentration measured during the course of the hospitalization. The primary study outcome was all cause mortality.

### BNP for screening and follow-up of PH

The use of BNP as an adjunct to echocardiography for screening and follow-up of BPD-associated PH has been adopted by our group since August 2010. ELBW infants who remained on oxygen and/or respiratory support at 28 days of age were evaluated with echocardiography and serum BNP measurement shortly thereafter (before 6 weeks of age) to screen for PH. Infants diagnosed with PH based on echocardiographic findings were then evaluated with monthly echocardiography and BNP testing for follow-up of severity of PH. Infants who did not show signs of PH on initial screening may be subsequently re-evaluated by echocardiography and BNP measurement based on clinical suspicion of PH. BNP levels were measured in pg/mL using the ADVIA Centaur® BNP Assay (Siemens USA).

Our general management strategy for PH in BPD infants utilizes a stepwise approach. Initial treatment included optimizing respiratory support to provide adequate oxygenation and prevent periods of hypoxemia. If oxygenation remains labile, pulmonary vasodilator therapy is added sequentially starting with inhaled nitric oxide, then sildenafil, and, for severe cases of PH, bosentan is sometimes considered.

### Statistical analyses

Values are presented as counts and percentages, mean ± standard deviation, or median and interquartile range (IQR, 25th and 75th percentile). Skewed data (BNP levels) were transformed logarithmically to produce a normal distribution for appropriate parametric testing. Comparison of baseline subject characteristics in survivors vs. non-survivors was performed with independent samples t-test, Mann–Whitney U, or Fisher’s exact test, as appropriate. Possible correlations between demographic and clinical variables and outcome were investigated using uni-and multivariate Cox proportional hazard analysis and hazard ratios with two-sided 95% confidence interval (CI) are provided. Before performing survival analysis, a receiver operating characteristic curve was created to determine the peak BNP level that provided the best combination of sensitivity and specificity for predicting the composite endpoint. Survival analysis was performed with Kaplan-Meier and Cox proportional hazards modeling. The log-rank test was used to determine statistical significance between Kaplan-Meier survival curves. BNP level, age, sex, and BPD severity were all included in the hazard model. All statistical tests were 2-sided, and P value of <0.05 was deemed significant.

## Results

### Baseline characteristics

During the study period, 36 preterm infants with BPD-associated PH had BNP values available. The mean gestational age was 26.0 ± 1.9 weeks and the mean birth weight was 740 ± 290 grams. The differences in characteristics between survivors and non-survivors are shown in Table 1. No differences were seen between survivors and non-survivors in terms of gestational age, birth weight, sex, or race. There were also no differences in terms of being small for gestational age (SGA), respiratory support at 36 weeks postmenstrual age and severity of BPD, severe intraventricular hemorrhage, or proven necrotizing enterocolitis. Of note, 6 of the 17 survivors (35%) were noted to have a PDA at the time of echocardiographic diagnosis of PH compared to none of the non-survivors (P = 0.006).

**Table 1:** Baseline and clinical characteristics for all patients, survivors and non-survivors

	**All patients**	**Survivors**	**Non-survivors**	**P value**
	**n = 36**	**n = 17**	**n = 19**	
Demographics				
Birth weight, grams	740 ± 290	745 ± 293	736 ± 295	0.92
Gender, Male	19 (53)	9 (53)	10 (53)	1.0
Race				0.86
Caucasian	15 (42)	7 (41)	8 (42)	
African American	20 (55)	9 (53)	11 (58)	
Hispanic	1 (3)	1 (6)	0 (0)	
Gestational age, weeks	26.0 ± 1.9	26.2 ± 2.4	25.8 ± 1.4	0.59
Small for gestational age	13 (36)	6 (35)	7 (37)	1.0
Respiratory support at 36 weeks postmenstrual age				0.62
Mechanical ventilation	15 (42)	8 (47)	7 (37)	
CPAP	5 (14)	1 (6)	4 (21)	
O_2_ supplementation	13 (36)	7 (41)	6 (32)	
Room air	3 (8)	1 (6)	2 (10)	
Morbidity				
BPD				0.61
Mild	3 (8)	1 (6)	2 (10.5)	
Moderate	2 (6)	0 (0)	2 (10.5)	
Severe	31 (86)	16 (94)	15 (79)	
Patent ductus arteriosus	6 (16)	6 (35)	0 (0)	0.006
Severe intraventricular hemorrhage (grade 3 or 4)	9 (25)	6 (35)	3 (8)	0.26
Proven necrotizing enterocolitis (stage 2 or 3)	5 (14)	4 (24)	1 (5)	0.17
Peak BNP, pg/mL	351 (82,1461)	128 (23,463)	997 (278,1770)	0.004
Medications				
Inhaled nitric oxide	32 (89)	13 (76)	19 (100)	0.04
Sildenafil	31 (86)	12 (71)	19 (100)	0.02
Bosentan	18 (50)	7 (41)	11 (58)	0.51

Data shown as *n* (%), mean ± standard deviation, or median (interquartile range). *P* values refer to independent samples t-test, Mann–Whitney U, or Fisher’s exact test.

*BNP* B-type natriuretic peptide, *BPD* bronchopulmonary dysplasia, *CPAP* continuous positive airway pressure, *O*_*2*_ oxygen.

### Relation of peak BNP and mortality from all causes

**Figure 1:**
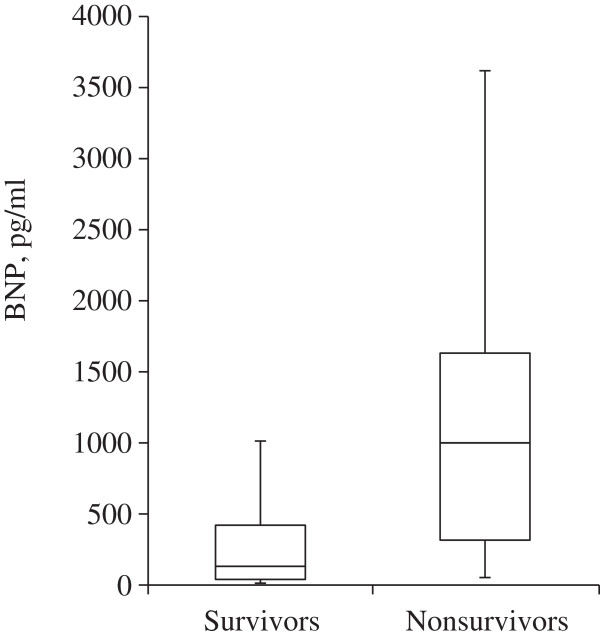
Box plots showing median levels of peak BNP of survivors versus non-survivors in a cohort of preterm infants with BPD-associated PH.

**Figure 2:**
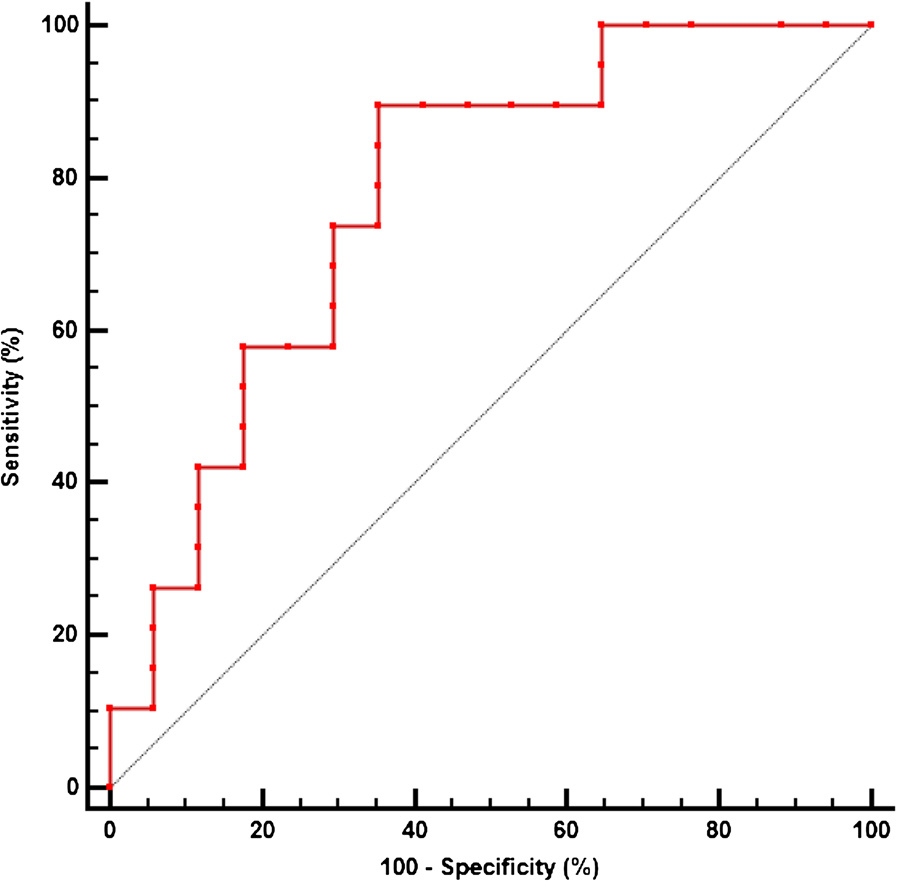
Receiver operating characteristic curve demonstrating that a BNP level of 220 pg/ml results in a sensitivity of 90% and a specificity of 65% for predicting mortality in preterm infants with BPD-associated PH.

The median peak BNP level was significantly lower among infants who survived than among those who died (128 pg/ml, [IQR 23 to 463] vs. 997 pg/ml, [IQR 278 to 1770], P < 0.004) (Figure 1). Analysis using a receiver operating characteristic curve shown in Figure 2 identified a peak BNP cutoff value of 220 pg/ml to have the best combination of sensitivity (90%) and specificity (65%) for predicting mortality from all causes.

**Figure 3:**
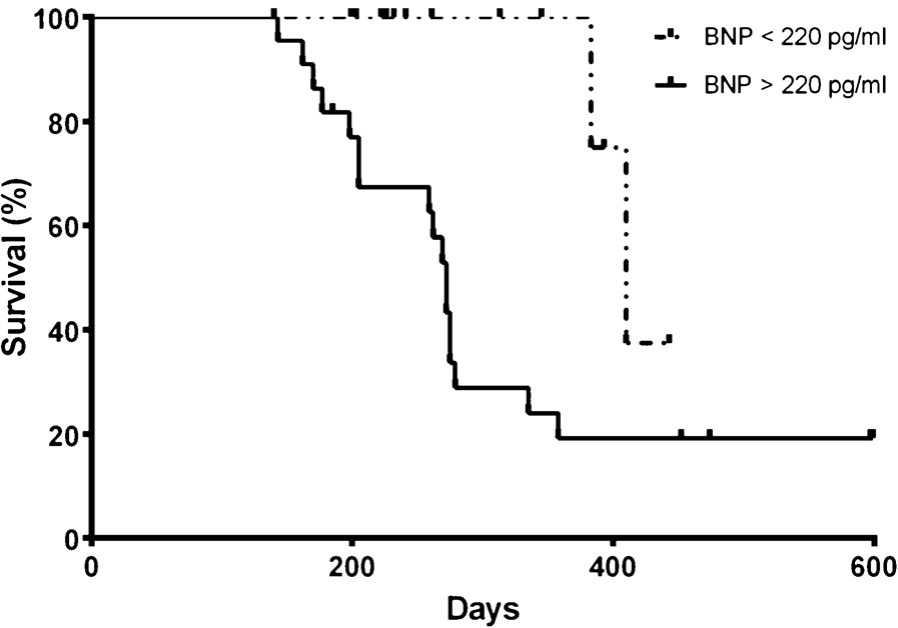
**Kaplan-Meier cumulative survival curves showing cumulative rates of survival for 36 preterm infants with BPD-associated PH stratified by the identified BNP cut-off value.** Infants with BNP ≥ 220 pg/ml differed significantly from infants with BNP < 220 pg/ml.

Comparison of baseline characteristics between subjects with peak BNP < 220 pg/ml and those with ≥ 220 pg/ml showed no significant differences. Kaplan-Meier survival analysis shown in Figure 3 demonstrated significantly lower survival for subjects with peak BNP level of ≥ 220 pg/ml After controlling for age, sex, and BPD severity, multivariate Cox proportional hazards modeling showed that a BNP level of ≥ 220 pg/ml independently increased the likelihood of death (hazard ratio 5.1, 95% CI 1.1 to 22.4, P = 0.03).

## Discussion

Our study demonstrates that peak BNP level obtained during the course of hospitalization in a cohort of preterm infants with BPD-associated PH provides prognostic information on all-cause mortality. This is consistent with previous studies suggesting that elevated BNP is associated with increased mortality in adult patients with various forms of PH [12,14,16]. Our group has also previously demonstrated that BNP levels may be useful in screening for PH in preterm infants with BPD [18]. The findings of this current study extend the clinical usefulness of BNP as a prognostic biomarker in preterm infants with BPD-associated PH.

Preterm infants with BPD-associated PH are difficult to manage. Availability of a non-invasive and readily available prognostic biomarker is beneficial for risk stratification and optimal management [19]. Identification of which infants are at increased risk and which are at low risk for adverse outcomes allows appropriate allocation of resources to infants who would benefit the most from specific treatment strategies, as well as prevent overtreatment of infants at low risk. Currently, echocardiography is used to determine severity of PH and assess risk for adverse outcomes. Though noninvasive and available in most centers, echocardiography is resource intensive and has limited sensitivity in determining severity of PH [20].

Our study suggests that elevated BNP levels may be useful for risk stratification in this vulnerable patient population. Our analysis was limited due to the small sample size and the fact that BNP measurements were not obtained at similar time points. Nevertheless, we did observe trends showing that BNP levels decreasing over time were seen among infants that survived; and that BNP levels increasing or remaining elevated over time were seen among infants who died. This observation needs further validation in a larger prospective study with well-defined BNP estimation time points, but it does suggest that serial BNP measurement may be helpful in identifying infants at high and low risk for mortality; and that treatment, including the intensity of surveillance and the use of aggressive pharmacologic and interventional therapy, may be adjusted accordingly.

More than one-third of infants with BPD-associated PH in our study were SGA. This is consistent with previous studies which reported that SGA infants are at an increased risk for developing PH [5,21]. It is interesting to note however that no difference in SGA status between survivors and non-survivors was seen in our study. This may indicate that despite the higher risk for developing PH, SGA infants are not necessarily at any greater risk for mortality compared to appropriate for gestational age infants with PH. Larger prospective studies will need to be carried out to validate this hypothesis.

It is also interesting to note that the presence of a PDA at the time of initial echocardiographic diagnosis of PH was noted in 6 of the 17 survivors versus none in those who died. We postulate that the presence of a PDA may be protective, allowing a “pop-off valve” that alleviates elevated pulmonary pressures [22,23]. Another difference is that more non-survivors were noted to be on inhaled nitric oxide or sildenafil than survivors. This most likely represents more severe disease among non-survivors requiring more intensive treatment for PH rather than any perceived harm from inhaled nitric oxide or sildenafil.

Four infants with peak BNP levels below the identified threshold of 220 pg/ml had late mortality, as reflected by a drop in their Kaplan-Meier survival curve at around 400 days of life. (Figure 3) Review of medical records indicate that the cause of death in 2 of these infants were due to disease processes not directly related to PH (sepsis, withdrawal of support for severe encephalopathy), which may explain why BNP levels remained below the threshold in these non-survivors. The remaining 2 infants however were identified to have died because of severe BPD and PH. This finding suggests that in a small subset of preterm infants, BNP levels may not rise as expected despite the presence of severe PH. Further studies are needed to validate this observation as well as identify characteristics that may determine which infants with PH fail to exhibit a rise in serum BNP.

The strengths of our study include its well defined cohort of preterm infants with BPD-associated PH and highly relevant primary outcome of mortality versus survival. Our study is limited by its small sample population, its retrospective nature, and the possibility that infants included are biased towards those with more severe disease. An important limitation is the lack of follow-up data on survivors, including rehospitalization and post-discharge mortality. Another limitation is our lack of data on renal function, as renal dysfunction is known to contribute to higher BNP levels [24,25]. A prospective study involving a larger sample size is needed to validate the usefulness of BNP as a prognostic marker in this population. Further studies are also needed to show whether treatment strategies guided by BNP levels will lead to decreased morbidity and mortality.

## Conclusion

Our findings suggest that elevated BNP levels in hospitalized preterm infants with BPD-associated PH may be used to identify infants at risk for death. BNP levels may be included in the risk stratification of preterm infants with PH, and higher BNP levels may warrant increased surveillance and management.

## Abbreviations

BNP: B-type natriuretic peptide; BPD: Bronchopulmonary dysplasia; CI: Confidence interval; ELBW: Extremely low birth weight; IQR: Interquartile range; PH: Pulmonary hypertension; PDA: Patent ductus arteriosus; SGA: Small for gestational age.

## Competing interests

The authors declare that they have no competing interests.

## Authors’ contributions

AC contributed to the design of the study, was responsible for the management and retrieval of data from the neonatal database, contributed to initial data analysis and interpretation, drafted the initial manuscript, and approved the final manuscript as submitted. JK was responsible for the management and retrieval of data from the neonatal database, contributed to initial data analysis and interpretation, and approved the final manuscript as submitted. BS conceptualized and designed the study, supervised all aspects of the study, critically reviewed and revised the manuscript, and approved the final manuscript as submitted. All authors read and approved the final manuscript.

## Pre-publication history

The pre-publication history for this paper can be accessed here:

http://www.biomedcentral.com/1471-2431/14/68/prepub
